# Assessing the Performance of Daily Intake of a Homotaurine, Carnosine, Forskolin, Vitamin B2, Vitamin B6, and Magnesium Based Food Supplement for the Maintenance of Visual Function in Patients with Primary Open Angle Glaucoma

**DOI:** 10.1155/2020/7879436

**Published:** 2020-01-17

**Authors:** Teresa Rolle, Laura Dallorto, Stefania Rossatto, Daniela Curto, Raffaele Nuzzi

**Affiliations:** Eye Clinic, Department of Surgical Sciences, University of Torino, Torino, Italy

## Abstract

**Background:**

Glaucoma is a multifactorial optic neuropathy, which causes a continuous loss of retinal ganglion cells. Given the neurodegenerative nature of glaucoma, the necessity for neuroprotective intervention still arises, to be added alongside hypotonic therapy.

**Objective:**

The objective of this study was to assess the effect of daily intake of a homotaurine, carnosine, forskolin, vitamins B1, B2, and B6, folic acid, and magnesium based supplement (GANGLIOLIFE®) on the progression rates of the visual field in patients with progressive POAG despite good tonometric compensation and to assess the most suitable dosage.

**Methods:**

This is a monocentric nonrandomized experimental clinical study. Patients with mean deviation (MD) ranging from −2 dB to −15 dB with MD progression ≥1 dB in the previous year and IOP values of ≤18 mm Hg were included. All the patients underwent supplement therapy for a period of 6 months. For the first 2 months, they took 2 tablets a day, and for the following 4 months, 1 tablet a day. The patients were assessed before the start of treatment, time 0 (*T*_0_), after 2 months (*T*_1_), and after 6 months (*T*_2_) of therapy. At each check-up, patients were given a full eye test including perimetry, RNFL, and GCC using FD-OCT, PERG, contrast sensitivity, and QoL evaluation using the Glaucoma Symptom Scale questionnaire and National Eye Institute Visual Function Questionnaire 25.

**Results:**

31 patients with a mean age of 70.80 ± 8.77 were included. At *T*_1_ and *T*_2_, the mean values of MD were lessened (MD = −5.37 ± −2.91, *P* < 0.01, and MD = −5.48 ± 3.15, *P* < 0.05, respectively) compared to *T*_0_ (MD = -5.98 ± 2.83). Patients also demonstrated a significant reduction in IOP (*P* < 0.01), improved light sensitivity (*P* < 0.01) and contrast sensitivity (*P* < 0.05), and a better quality of life (*P* < 0.05).

**Conclusions:**

Treatment with a supplement which includes homotaurine, carnosine, forskolin, vitamins B1, B2, and B6, folic acid, and magnesium has been shown to be able to slow down the rate of progression of functional damage and improve visual function after 2 and 6 months of daily intake. Quality of life showed significant improvement.

## 1. Introduction

Glaucoma is a multifactorial optic neuropathy leading to a continuous loss of retinal ganglion cells [1, 2]. The loss of the visual field in glaucoma is actually the direct consequence of the loss of retinal nerve fibres, which have a specific anatomical arrangement in the retina: the nerve fibres of the temporal retina do not cross the horizontal median line and, therefore, the scotomas follow the shape of these bands of fibres, giving rise to defects in the visual field characteristic of glaucoma (arcuate or arciform scotomas) [3, 4].

Recent discoveries have led to a new perception of glaucomatous pathology: the term Glaucoma 2.0 [2] refers to the recent attention paid to neuronal damage and to the neurodegenerative nature of the disease, which has provided the foundation for a new generation of therapeutic targets.

As further proof of its neurodegenerative nature, primary open-angle glaucoma presents important analogies with other neurodegenerative diseases: it has in fact been shown that retinal ganglion cells (RCG) have cell death mechanisms similar to those of Alzheimer's disease (AD), primarily represented by apoptosis (programmed cell death). Among the mechanisms capable of triggering the apoptotic cascade in glaucoma is protein misfolding. *β*-Amyloid deposits, which are characteristic of Alzheimer's disease, have, in fact, recently been implicated in the pathogenesis of glaucoma [5]. Many studies in the literature [5–10] have investigated the correlation between these two diseases, coming to the conclusion that, in addition to sharing pathogenic mechanisms, the incidence of glaucoma is comparatively higher in patients with Alzheimer's disease as compared to controls of the same age: 26% versus 5% in a German population [6] and 24% versus 9% in a Japanese sample [7]. Furthermore, the progression of visual field defects is accelerated in patients with open-angle glaucoma and AD compared to patients with open-angle glaucoma without AD [10].

Glaucoma is not only a pathology of the eye but a pathology of the central nervous system [11]: in fact, cell death also affects, in addition to the retinal ganglion cells, the neurons of the lateral geniculate body, the area of the encephalic thalamus responsible for the elaboration of visual information, and those of the visual cerebral cortex [12, 13].

These opinions are supported by a recent review [14] that by means of modern techniques of neuroimaging has objectified the anomalies along the visual and extravisual pathway and in the interconnections between these different areas in patients suffering from POAG.

The neurodegenerative nature of the glaucomatous pathology makes a neuroprotective intervention aimed at the survival of retinal ganglion cells necessary, in addition to the lowering of intraocular pressure, which is the main risk factor of the disease. The reduction of IOP does not always mean control of the disease; the main studies on antiglaucomatous therapy (EMGT and OHTS) [15, 16] have demonstrated an advancement of the damage despite pressure control. Moreover, the existence of low pressure glaucoma (NTG) is well documented, in which the damage to the optic nerve does not come along with a pressure increase [17].

The most rational strategy to be followed for neuroprotective treatment is that of simultaneously counteracting the principal neurodegenerative mechanisms, in order to slow down the progression of the glaucomatous disease [18, 19]. Specifically, three different approaches are suggested: neuroprotection, neuroregeneration, and neuroenhancement [2]. Neuroprotection involves slowing down and preventing neuronal death to maintain physiological functions. Neuroregeneration aims to encourage neuronal regeneration and reconstruct the connections from the eye to the brain in axons that have already been damaged. Finally, the last proposed approach is that of neuroenhancement, literally of neuro-empowerment, which endeavours to improve the functioning of the ganglion cells that are affected and have lower performances but are still alive. In fact, some studies performed by means of a pattern electroretinogram (PERG) have objectified a reversible dysfunction after the reduction of the IOP and response anomalies that precede the damage of the visual field [20, 21].

Within the field of these three approaches aimed at ganglion cells, many molecules have been studied, including Citicoline, *Gingko biloba*, palmitoylethanolamide, homotaurine, carnosine, and forskolin. The role of some of these substances has already been demonstrated, and they have already entered clinical practice, while others have scientific underpinnings but still require further clinical efficacy studies.

The molecules contained in the supplement which is the subject of the study are magnesium, *Coleus forskohlii* extract, homotaurine, L-carnosine, vitamins B1, B2, and B6, and folic acid. Homotaurine is a substance extracted from marine algae. It is a low-molecular weight sulphonate compound capable of binding to a *β*-peptide (the pathological element typical of Alzheimer's and of brain aging) in its soluble form [22]. Homotaurine also has a direct effect on neuronal activity; thanks to its affinity with GABA A receptors, it modulates cortical inhibitory activity by reducing the response of neurons to excitatory stimuli to glutamate [18, 23–27]. Carnosine, by improving mitochondrial respiration, prevents the formation of free oxygen radicals and can significantly counteract oxidative stress [28, 29]. Forskolin, by activating the adenylate cyclase enzyme, causes an increase in the intracellular levels of cyclic AMP [30–32], with a subsequent increase in the production of neurotrophins [33, 34]. Forskolin has also been shown to lower IOP by reducing the production of aqueous humour [35, 36]. It is on this basis that the supplement GANGLIOLIFE® also claims to have a lowering role on IOP. Folic acid also seems to play a role in the prevention of glaucoma due to its ability to counter hyperhomocysteinemia, implicated among the various pathogenic factors of damage [37, 38].

The study we conducted therefore fits into the context of new scientific evidence that the neurological abnormalities found in patients with POAG are not limited to retinal ganglion cells but extend to the whole visual pathway and to extravisual areas, making glaucoma a diffuse neurodegenerative disease [14].

The primary objective was to evaluate the effect of treatment with a homotaurine, carnosine, forskolin, vitamins B2, vitamin B6, and magnesium based food supplement on the rate of progression of the disease (change of mean deviation at the visual field test/year) and to study the best dosage. As secondary end points, the study also proposed the variations of structural damage (expressed as variation of RNFL and annual GCC evaluated by OCT), the reduction of IOP, and the tolerability of the supplement, monitoring the appearance of possible side effects. Finally, quality of life before and after treatment was also assessed.

## 2. Materials and Methods

In this monocentric nonrandomized clinical study, we have included patients who consecutively presented at the Glaucoma Clinic of the University of Turin Eye Clinic between January 2016 and December 2016. The glaucoma center of the University of Turin is one of the biggest Glaucoma Center of the Piedmont (region of North-Western Italy). It represents the reference for about one-third of the 4.5 million people living in Piedmont. Thus, we can assume that the cohort of patient selected for this study is representative of the glaucomatous patients in that area of Italy.

The study was approved by the Local Ethics Committee (Ethics Intercompany Committee A.O.U. City of Health and Science of Turin-A.O. Ordine Mauriziano-A.S.L. City of Turin) and was conducted in accordance with the principles of the Helsinki Declaration. Before initiating the treatment, every patient enrolled in the study provided their informed written consent.

In order to be included, the patients had to be between 18 and 85 years of age, present Humphrey 24-2 SITA standard visual field that is reliable, and with glaucomatous alterations according to Hodapp criteria; have mean deviation (MD) between −2 dB and −15 dB; have alterations of the head of the optic nerve such as incisures of the neuroretinal rim, asymmetry of the cup-disc relationship between the two eyes, disc hemorrhages, and diffuse or localized defects of the retinal nerve fibre layer; and have MD progression ≥1 dB per year in the previous year with IOP values ≤18 mmHg in antiglaucomatous therapy.

Patients with visual acuity of less than 0.5, equivalent spherical refractive defect greater than +3 diopters or less than −6 diopters, presence of other ocular pathologies, other forms of glaucoma, previous ocular surgery performed in the last 6 months, dysmetabolic and neurological pathologies that may cause changes in the visual field or optical disc, treatment with Brimonidina in monotherapy and/or in association, poor collaboration on clinical examination, and presumed or confirmed pregnancy status were excluded.

### 2.1. Treatment with Supplement

The treatment using GANGLIOLIFE® (GLAUCOOM—Glaucoma Division SOOFT Italia spa, Montegiorgio, Italy), consisting of 150 mg of *Coleus forskohlii* dry root extract, 10% of forskolin, 100 mg of homotaurine, 50 mg of L-carnosine, 0.2 mg of folic acid, 150 mg of magnesium, 1.1 mg of vitamin B1, 1.4 mg of vitamin B2, and 1.4 mg of Vitamin B6, was divided into two stages. During the first period, the treatment consisted of 2 tablets a day, one in the morning and one in the evening, before meals, for 60 days. The second period consists of the administration of 1 tablet a day, in the morning before meals, for the next 120 days. Patients continued topical hypotonic therapy for the duration of the study.

### 2.2. Examinations

Patients were assessed before the start of treatment, time 0 (*T*_0_), and after 2 months (*T*_1_) and 6 months (*T*_2_) of therapy. At time 0, medical history was taken. At each visit, the patients underwent a complete ophthalmological examination including Goldmann applanation tonometry and standardized automated perimetry using a Humphrey Field Analyzer (HFA) (Carl Zeiss Meditec, Jena, Germany) using the standard Swedish interactive threshold algorithm strategy (SITA), program 24-2. To be considered reliable, the visual field had to have a number of false positives and negatives <33% and fixation losses <20%.

All subjects underwent the entire FD-OCT RTVue-100 (Optovue Inc, Fremont, CA) Glaucoma protocol, which includes the 3D-disc, ONH, and GCC scans. Both eyes of all subjects were examined three times, always by the same operator. The thickness of the RNFL was determined by the ONH scan consisting of thirteen concentric linear scans centred on the optical disc, with diameters ranging from 1.3 to 4.9 mm. These scans are used to create a map of the thickness of the peripapillary nerve fibres (RNFL), while twelve radial scans of 3.7 mm in length make it possible to determine the morphological parameters of the disc margin. The scan of the three layers of the ganglion cell complex (GCC) covers an area of 7 × 7 mm centred on the fovea. Scans with motion artefacts and with a signal intensity index (SSI) of less than 45 were excluded from the study. Patients also underwent a pattern electroretinogram (PERG) registered with Retimax (C.S.O. srl, Italy) at a spatial frequency of 60′, respecting ISCEV standards, and evaluation of contrast sensitivity using sine-wave gratings. QoL was evaluated by giving patients the Glaucoma Symptom Scale (GSS) questionnaire and the National Eye Institute Visual Function Questionnaire 25 (NEI-VFQ 25). The answers of the NEI-VFQ-25 questionnaire were divided into 12 precoded subscales that covered the fields of general health (GH), general vision (GV), ocular pain (OP), difficulties in activities that require near vision (NA), difficulties in activities that require a vision from afar (difficulty with distance vision activities, DA), limitations in relationships due to vision (limitations in social functioning due to vision, VSSF), role limitations due to vision (VSRD), dependency on others due to vision (VSD), psychic visual symptoms due to vision (mental health symptoms due to vision, VSMH), driving difficulties (D), and limitations in peripheral and colour vision (limitations with peripheral, PV and colour vision, CV). For every subscale, the average score of the sum of the questions was calculated.

### 2.3. Statistical Analysis

The numerosity of the subject sample of our study was evaluated using the following numerosity estimation formula for the comparison of two means. The null hypothesis is that *m*_1_ = *m*_2_ (*m*_1_ is the mean of the progression rates before treatment and *m*_2_ is the mean of the progression rates after taking GANGLIOLIFE®); the alternative hypothesis is *m*_1_ = *m*_2_ + *d*, where *d* is the difference between the two means:

(1) $$\begin{array}{c} N=\frac{\left(r+1\right){\left(Z_{\alpha /2}+Z_{1-\beta }\right)}^{2}s^{2}}{rd^{2}}, \end{array}$$

where *Z*_*α*/2_ = the value of *Z* for two-tail confidence intervals (*Z* = 1.96 for 95% confidence interval), *Z*_1−*β*_ is equal to 0.84 for a power of 80%, *r* = *n*_1_/*n*_2_ is equal to 1 because the two samples have the same numerosity, *s* is the estimated standard deviation, which was hypothesized to be 0.7 dB/year, based on studies on the progression of glaucomatous damage to the visual field [9], and *d* is the difference between the minimum means considered significant.

As we do not have any previous studies on the effect of the association of homotaurine, carnosine, and other elements on the rate of progression of glaucoma, we assumed a 50% reduction in the annual progression rate as a possible neuroprotective effect of the supplement (*d* = 1 db/year − 0.5 = 0.5).

Based on this data, the sample size is 30 people.

The data was collected and processed using the programme Microsoft Office Excel and SPSS statistical programme for Windows, version 19.0, SPSS Inc, Chicago, IL. The continuous variables respected normal distribution according to the Shapiro–Wilk test. The mean results were represented as means and relative standard deviations. The mean results of the various parameters obtained at time 0 were compared with the results at time 1, after two months of therapy, and at time 2, after 6 months of therapy, using Student's *t*-test for dependent samples.

The primary outcome of the study was the rate of progression (change in MD/year). The rate of progression in the year before entering the study was compared with the rate of progression after the beginning of neuroprotective therapy. Our study had a follow-up of 6 months, so the rate of progression after one year is an estimated value. Mean slopes of change were calculated and compared. All the other parameters are secondary outcomes.

Possible influences on the rate of progression by variables such as age, type of treatment, IOP, and CCT were investigated by linear regression and Pearson correlation.

A significance level of *P* < 0.05 was adopted for each analysis. Where there were multiple comparisons, the Bonferroni correction was applied.

## 3. Results

Forty-five patients met the inclusion criteria. Of these, 13 were not included due to the poor quality of instrumental examinations given their lack of collaboration and one patient left the study early because of gastrointestinal side effects. Therefore, a total of 31 patients with primary open-angle glaucoma (POAG) were included in the study with a mean age of 70.80 ± 8.77 years (range 40–85 years). Demographic and clinical characteristics before initiating the treatment are listed in Table 1.

At the beginning of the study, the IOP median was 15.73 ± 2.11 mmHg. At 2 and 6 months of the treatment, IOP was statistically significantly reduced (IOP = 13.92 ± 2.91 mmHg and 14.43 ± 2.84 mmHg respectively).

Analysis of mean deviation values of the visual field and rate of progression which was the primary outcome of the study showed a significant improvement of MD both at *T*_1_ (MD = −5.37 ± 2.91, *P* < 0.05) and at *T*_2_ (MD = −5.48 ± 3.15, *P*=0.018) compared to the baseline (Table 2). The mean rate of progression before entering the study was −2.05 ± 1.67 dB/year. The mean rate of progression estimated on the basis of the 6-month study was 1.00 ± 2.87 dB/year. Therefore, no further progression of the damage was observed. In addition, MD values show an improvement of 0.61 ± 1.29 dB after 2 months of therapy and 0.5 ± 1.29 dB after 6 months of therapy.

Table 3 shows the average values, at the various times, of light sensitivity expressed in logarithmic units (dB). Total light sensitivity both at *T*_1_ (*P*=0.006) and *T*_2_ (*P*=0.001), superior hemifield light sensitivity (*T*_1_, *P*=0.004; *T*_2_, *P*=0.001), inferior hemifield light sensitivity (*T*_2_, *P*=0.019), and sensitivity of the 16 central points (*T*_1_*P*=0.006) are significantly improved.

No significant variations were noticed with regard to RNFL and GCC as measured by FD-OCT.

Table 4 shows the average variations of the amplitude and latency values recorded with PERGs. We found a reduction in latency throughout the duration of therapy, particularly between *T*_0_ and *T*_2_ and between *T*_1_ and *T*_2_ (*P*=0.03) and an increase in amplitude between *T*_0_ and *T*_2_ that was not statistically significant (*P*=0.03).

Analysis of the NEI-VFQ-25 (National Eye Institute Visual Function Questionnaire 25) showed a significant improvement in quality of vision in the comparison between *T*_0_ and *T*_1_ (*P*=0.0003), between *T*_1_ and *T*_2_ (*P*=0.022), and between *T*_0_ and *T*_2_ (*P*=0.011). With regard to overall quality of life, 84% of patients at *T*_1_ can be defined as improved, 8% worsened, and 8% constant.

The answers of the NEI-VFQ-25 questionnaire were subdivided into the 12 precoded subscales (Table 5). For each subscale, we compared the values between pretreatment and posttreatment. We obtained a significant value of *P* < 0.05 for most of the subscales, in particular, between *T*_0_ and *T*_1_.

With regard to symptomatology, using the Glaucoma Symptom Scale (GSS), a mean score improvement was found between *T*_0_ and *T*_1_ (*P*=0.001), between *T*_1_ and *T*_2_ (*P*=0.001), and between *T*_0_ and *T*_2_ (*P*=0.002), as shown in Table 6. On the basis of GSS results, 56% of patients improved their symptomatology at *T*_1_, 13% worsened it, and in 31% of the total, it remained constant. Specifically, the following symptoms were improved: burning, stinging sensation, tearing, sensation of dryness, and difficulty seeing in daylight and in dark places.

## 4. Discussion

This study was carried out in order to assess the effect of the intake of a supplement containing homotaurine, carnosine, *Coleus forskohlii*, vitamins B1, B2, and B6, folic acid, and magnesium (GANGLIOLIFE®) on the structural and functional progression of the glaucomatous damage.

In agreement with literature data showing how the mechanisms of action of these substances interfere with the different mechanisms of the pathogenesis and progression of glaucoma, we observed an important improvement in the rates of progression of the perimetric damage, expressed as MD/year, and of the structural and functional damage in the period following the intake of the supplement.

However, the perimetric damage is prominent when a significant amount of ganglion cells have been damaged, depending on the studies, in a percentage ranging between 25 and 50% [39–43]. Therefore, other methods such as PERG could be better candidates to see changes in ganglion cell responses in the short term. In glaucoma patients treated with the oral supplement, we observed an improvement in ganglion cell response with increased amplitude and reduced latency. This improvement could also be because of the reduction of IOP, as demonstrated in the literature [21, 44]. Improvement in bioelectrical retinal responses with PERG was also demonstrated after a 12-month treatment period with Coenzyme Q10 and Vitamin E in eye drops [45].

As expected, our results supported the reduction of IOP in accordance with scientific studies that demonstrate the antihypertensive effects of forskolin [36].

As far as the dosage is concerned, significant results were obtained with the administration of 2 tablets a day. The objective of this study was to assess the best dosage to obtain and maintain the best benefits. The protocol with 2 tablets for the first two months, and then the protocol with 1 tablet was chosen to evaluate the potential possibility of reducing therapy after a first phase of “attack dose.” The dosage change to a single tablet after 60 days showed a slight reduction in the effects on the advancement of the damage. The study performed by Mutolo et al. [46] on the effects after administration of the same supplement showed significant results only after 6 months of therapy with 2 tablets a day.

Our study highlights a statistically significant improvement in the quality of life of glaucoma patients demonstrated through the most commonly used questionnaires in this type of patient, NEI-VFQ-25 and GSS. In particular, with the NEI-VFQ-25, a statistically significant improvement was highlighted in the categories of general vision, eye pain, near vision activities, visuospecific social function, visuospecific mental health, and peripheral vision.

The possible mechanisms of action of the various components of the supplement can be explained on the basis of experimental studies. Homotaurine links with the soluble form of beta-amyloid that can no longer assume fibrillar structure (non-soluble form) and would favour its elimination, thereby preventing its accumulation. By binding to the soluble amyloid protein, it prevents the aggregation and formation of amyloid plaques also found at the level of retinal ganglion cells (RGCs) in primary open-angle glaucoma responsible for cell death [22, 23]. Homotaurine also counteracts excitotoxic damage, that is, the pathogenic mechanism through which the RGC and other nerve cells die as a consequence of an excess of extracellular glutamate [18]. Glutamate, by binding to glutamate receptors, of which the N-methyl-D-aspartate (NMDA) receptor represents the main exponent, causes an influx of Ca^2+^ in the cell. The excess of glutamate would therefore determine an excess of intracellular Ca^2+^ and the activation of a proapoptotic signal. The role of homotaurine in cortical changes in GABAergic transmission has also been tested *in vivo* [24]. Studies conducted in patients with AD have shown that homotaurine can reduce cerebrospinal A*β*1-42 levels in patients with Alzheimer's disease [25], reduce cerebral atrophy [26] and have positive cognitive effects [27]. The effects of Homotaurine on neuroprotection in glaucomatous disease have not yet been investigated by clinical studies from the published literature. However, the results on Alzheimer's disease and the similarities between the latter and glaucoma make homotaurine an excellent candidate for antiglaucomatous neuroprotective therapy.

The use of carnosine is based on the evidence that it counteracts oxidative stress. The latter phenomenon plays a significant role in neuronal cell death and in the death of RGC in glaucoma, as demonstrated by experimental models of glaucoma and by *in vivo* studies [30–32]. Furthermore, carnosine can prevent an increase of glutamate concentrations, reducing the glutamate excitotoxicity phenomenon [29]. The ability of Carnosine in double formulation (systemic and oral) to reduce oxidative stress in glaucoma related trabecula [33] has emerged in a recent study.

Forskolin increases the levels of neurotrophins, particularly BDNF (Brain Derived Neurotrophic Factor), since the deprivation of neurotrophins is one of the causes of apoptosis of RGCs [35].

Results similar to ours came from other studies carried out with molecules using similar mechanism of action and on a sample of subjects similar to that of our study, showing efficacy in the reduction of glaucomatous progression. Specifically, one of the most studied molecules in the field of glaucoma neuroprotection is citicoline. A recent study by Ottobelli et al. [47] demonstrates how the intake of oral citicoline for two years reduces the progression rate of glaucomatous disease (from −1.1 db/year to −0.15 db/year). The neuroprotective role of citicoline has also been proven in a trial study on citicoline in eye drops conducted by Roberti et al. [48].

*Ginkgo biloba* extract has also been tested in the treatment of low-tension glaucoma: in some patients, it seems to improve the preexisting damage to the visual field [49]. Its extract (EGb761), which has flavonoids and terpenoids, in fact, has been shown to lower the loss of RGC. There are many pharmacological properties: antioxidant effects moderating the release of electrons to free radicals, stabilization of the mitochondrial membrane by restoring the respiratory chain, increasing the production of ATP, and increasing vasodilatation [50]. Lastly, we mention the effects of palmitoylethanolamide (PEA) both on progression of damage to the visual field and on intraocular pressure [51–53]. A study done in Italy [51] showed a reduction in the rates of visual field progression both in terms of mean deviation and pattern standard deviation in the group of subjects with normal pressure glaucoma who took the PEA as compared to the control group taking a placebo.

Therefore, the abundant and well-established scientific evidence on the effectiveness of neuroprotection in glaucoma has led to the inclusion of neuroprotection among the therapeutic options in the latest edition of the guidelines of the European Glaucoma Society [1].

The study has its limitations. The supplement presents different molecules, each with a different mechanism of action, so it is difficult to establish the exact role of each one. The data should be compared with a control group with the same characteristics and the same decrease of IOP to exclude the sole effect of lowering IOP on the results obtained. Nevertheless, a randomized clinical trial (RCT) would be methodologically more correct for evaluating treatment effect. However, a RCT with a group of patients receiving placebo would raise some ethical concerns since there are evidences that the neuroprotective treatment slows down the progression of the glaucomatous disease.

In addition, our study reports initial data at 6 months; studies with a longer follow-up are desirable. Due to the 6-month follow-up, the rate of progression expressed as dB/year is an estimated value and this represents the biggest limitation of our study.

Finally, our study demonstrates an important role for the homotaurine, carnosine, forskolin, vitamins B2 and B6, and magnesium based supplement in the field of neuroprotection, which leads to an improvement in the patient's quality of life. The supplement subject of the study can therefore be considered a useful complementary therapy, associated with hypotensive therapy, in glaucomatous patients.

## Figures and Tables

**Table 1:** Demographic characteristics and clinical parameters at the baseline.

Parameters	Mean ± DS
No. of patients	31
No. of eyes	49
Sex, M/F (%)	35.5/64.5%
Age (years)	70.80 ± 8.77
Visual acuity (D)	0.8 ± 0.17
Spherical equivalent (D)	−0.4 ± 1.90
IOP (mmHg)	15.73 ± 2.11
CCT	558.35 ± 35.34
MD	−5.98 ± 2.83
PSD	6.05 ± 3.26
RoP (dB/year)	−2.05 ± 1.67

D = diopters; IOP = intraocular pressure; CCT = central corneal thickness; MD = mean deviation; PSD = pattern standard deviation; RoP = rate of progression.

**Table 2:** Differences of rate of progression and perimetric values expressed in MD at time 0 (*T*_0_), after 60 days (*T*_1_), and after 180 days (*T*_2_) of therapy.

Rate of progression						

		Before the treatment		After the treatment	*P*	
Mean		−2.05 dB/year		1.00 dB/year	<0.001^*∗*^	
DS		1.67 dB/year		2.87 dB/year		

Mean deviation						

	*T*_0_	*T*_1_	*T*_2_	*P T*_0_–*T*_1_	*P T*_0_–*T*_2_	*P T*_1_–*T*_2_
Mean	−5.98 dB	−5.37 dB	−5.48 dB	0.002^*∗*^	0.018^*∗*^	0.021^*∗*^
DS	2.83 dB	2.91 dB	3.15 dB			

^*∗*^Significant statistical values.

**Table 3:** Differences of light sensitivity at time 0 (*T*_0_), after 60 days (*T*_1_) and after 180 days (*T*_2_) of therapy.

Light Sensitivity (LS)	*T*_0_ mean ± DS	*T*_1_ mean ± DS	*T*_2_ mean ± DS	*P T*_0_ vs. *T*_1_	*P T*_0_ vs. *T*_2_
Total LS (DB)	23.24 ± 2.76	23.82 ± 2.86	23.96 ± 2.96	0.006^*∗*^	0.001^*∗*^
Superior hemifield LS (DB)	21.49 ± 4.55	22.25 ± 4.44	22.31 ± 4.38	0.004^*∗*^	0.001^*∗*^
Inferior hemifield LS (DB)	24.99 ± 3.45	25.40 ± 3.34	25.61 ± 3.76	0.12	0.019^*∗*^
16 central points LS (DB)	24.63 ± 3.85	25.29 ± 3.69	24.99 ± 4.07	0.006^*∗*^	0.13

^*∗*^Significant statistical values.

**Table 4:** Mean electrophysiological values observed at time 0 (*T*_0_), after 60 days (*T*_1_) and after 180 days (*T*_2_) of therapy.

PERG						
	*T*_0_	*T*_1_	*T*_2_	*P T*_0_–*T*_1_	*P T*_0_–*T*_2_	*P T*_1_–*T*_2_
Amplitude P50 (*µ*V)	3.54 ± 1.76	3.24 ± 1.56	3.87 ± 1.39	0.36	0.03^*∗*^	0.26
Latency P50-N95 (ms)	58.67 ± 7.67	57.46 ± 8.03	55.69 ± 5.47	0.30	0.30	0.03^*∗*^

^*∗*^Significant statistical values.

**Table 5:** Results of the National Eye Institute Visual Function Questionnaire 25 (NEI-VFQ 25).

	*T*_0_	*T*_1_	*T*_2_	*P T*_0_–*T*_1_	*P T*_1_–*T*_2_	*P T*_0_–*T*_2_
Total						
Mean	81.03	84.63	83.32	0.0003^*∗*^	0.0220^*∗*^	0.0109^*∗*^
DS	11.06	8.78	10.17			

General health						
Mean	130.80	143.00	130.60	0.0173^*∗*^	0.0028^*∗*^	0.9637
DS	27.64	31.66	33.24			

General vision						
Mean	122.80	136.40	132.80	0.0002^*∗*^	0.4124	0.0197^*∗*^
DS	21.70	21.39	25.25			

Ocular pain						
Mean	149.00	158.00	159.00	0.0094^*∗*^	0.7136	0.0384^*∗*^
DS	32.66	32.85	36.00			

Near activities						
Mean	453.00	498.00	483.00	0.0001^*∗*^	0.1261	0.0212^*∗*^
DS	111.18	80.01	88.01			

Distance activities						
Mean	494.00	522.00	508.00	0.0033^*∗*^	0.0076^*∗*^	0.1339
DS	90.51	87.60	90.06			

Visuospecific social function						
Mean	275.00	285.00	286.00	0.0151^*∗*^	0.8571	0.0457^*∗*^
DS	38.19	33.07	28.94			

Visuospecific mental health						
Mean	378.00	399.00	396.00	0.0244^*∗*^	0.6406	0.0211^*∗*^
DS	71.92	66.33	73.84			

Difficulty of role						
Mean	371.00	368.00	364.00	0.6116	0.4440	0.1479
DS	40.62	40.52	42.74			

Dependency						
Mean	379.00	384.00	380.00	0.2596	0.0429^*∗*^	0.8022
DS	32.82	30.52	32.27			

Guide						
Mean	181.00	184.00	196.00	0.2596	0.0761	0.0655
DS	99.29	98.66	96.47			

Colour vision						
Mean	92.00	96.00	95.00	0.1034	0.5743	0.3273
DS	18.71	15.61	16.14			

Peripheral vision						
Mean	79.00	85.00	84.00	0.0559	0.6639	0.0220^*∗*^
DS	27.65	21.65	23.80			

^*∗*^Significant statistical values.

**Table 6:** Results of Glaucoma Symptom Scale (GSS) Questionnaire.

	*T*_0_	*T*_1_	*T*_2_	*P T*_0_–*T*_1_	*P T*_1_–*T*_2_	*P T*_0_–*T*_2_
Total						
Mean	72.65	75.40	76.25	0.001^*∗*^	0.001^*∗*^	0.002^*∗*^
DS	20.63	19.09	20.69			

Question 1						
Mean	72.50	77.00	77.00	0.02^*∗*^	0.03^*∗*^	0.05
DS	32.44	29.81	31.88			

Question 2						
Mean	76.00	81.50	75.00	0.01^*∗*^	0.01^*∗*^	0.03^*∗*^
DS	31.53	29.37	33.50			

Question 3						
Mean	63.00	68.50	73.50	0.01^*∗*^	0.01^*∗*^	0.01^*∗*^
DS	37.88	32.27	36.21			

Question 4						
Mean	77.00	78.00	80.50	0.25	0.09	0.09
DS	29.38	25.58	25.90			

Question 5						
Media	70.00	70.00	74.00	0.77	0.72	0.09
DS	31.13	29.45	31.93			

Question 6						
Mean	70.50	73.50	76.50	0.07	0.12	0.17
DS	34.51	31.30	31.71			

Question 7						
Mean	78	79	77	1.00	0.99	1.00
DS	31.80	31.28	31.48			

Question 8						
Mean	77.50	81.00	83.00	0.01^*∗*^	0.02^*∗*^	0.05^*∗*^
DS	36.51	31.77	32.51			

Question 9						
Mean	63.50	67.00	68.50	0.02^*∗*^	0.03^*∗*^	0.09
DS	36.50	31.72	34.92			

Question 10						
Mean	78.50	78.50	77.50	0.77	0.77	0.75
DS	33.51	31.14	32.83			

^*∗*^Significant statistical values. Question 1: burning, stinging sensation. Question 2: tearing. Question 3: dryness. Question 4: itching. Question 5: pain, dryness/eyestrain. Question 6: cloudy/blurred vision. Question 7: sensation of a foreign body. Question 8: difficulty seeing in the light of day. Question 9: difficulty seeing in dark places. Question 10: perception of halos around lights.

## Data Availability

The Excel table data used to support the findings of this study are available from the corresponding author upon request.
